# Pathological characteristics and genetic features of melanin-producing medullary thyroid carcinoma

**DOI:** 10.1186/s13000-018-0764-2

**Published:** 2018-11-13

**Authors:** Changsong Wang, Tian Yun, Zhicheng Wang, Nianlong Meng, Naijun Fan, Xuexia Lv, Fulin Li

**Affiliations:** 1 Department of Pathology, 150th Hospital of PLA, Luoyang, Henan 471000 People’s Republic of China; 2 Department of Pathology, 153th Hospital of PLA, Zhengzhou, Henan 450042 People’s Republic of China

**Keywords:** Medullary thyroid carcinoma, Melanin, Immunohistochemistry, Diagnosis, Genetic feature

## Abstract

**Objective:**

To study the clinicopathological characteristics and genetic features of melanin-producing medullary thyroid carcinoma (MP-MTC).

**Methods:**

The immunophenotype of MP-MTC was studied using the immunohistochemical method, and its genetic features were assayed using an amplification refractory mutation system or PCR method.

**Results:**

A 71-year-old man presented with a slowly growing 5-cm mass on the left side of the neck for approximately two months. The cut surface of the neoplasm was brown and black. Melanin was found in the cytoplasm of tumor cells or the extracellular matrix. The tumor cells were positive for AE1/AE3, S-100 protein, melan A, HMB-45, synaptophysin, calcitonin, chromogranin A, melanoma, and thyroid transcription factor-1 (TTF-1) and negative for thyroglobulin. No typical genetic features were observed in this case. The patient showed no symptoms and recurrence at 12 months after the operation.

**Conclusions:**

The tumor cells of MP-MTC were positive for melanin biomarkers, TTF-1 and exhibited no genetic features. Histopathology and immunohistochemistry of the tumor cells will aid accurate diagnosis.

## Background

Melanin-producing medullary thyroid carcinoma (MTC) is an extremely rare MTC subtype. This malignant tumor of the thyroid originates from the C cells of the thyroid and displays varied cytological features and growth patterns. Several MTC subtypes, such as papillary/pseudopapillary, eosinophil, spindle cell, follicular, clear cell, giant cell, squamous cell, hemangiosarcoma, and paraganglioma-like subtypes, have been reported in the literature [1], but only a few reports on melanin-producing MTC have been published. According to the 2008 WHO tumor classification, melanin in MTC can range from microscopic foci to massive production, whereas MTC that produces large amounts of melanin is extremely rare. In the present report, we presented a case of MTC producing massive amounts of melanin. Given that this casehave yet to be reported in the literature, the genetic features of this type of MTC were studied at the same time.

## Methods and results

### Clinical summary

A 71-year-old male presented a slowly growing 5 cm mass on the left side of the neck for approximately two months. At first, the mass almost was not detectable through the naked eye. The mass increased in size to 5 cm in one month and was painful when applied with hand pressure. Physical examination indicated the hard mass size of 5 cm × 4 cm. The patient exhibited no clinical symptoms, such as face flushing, cardiovascular disturbances, and/or diarrhea. His past medical and family histories were unremarkable. The patient presented no history of infective diseases. Laboratory examination showed no abnormalities, and the thyroid hormonal index was within normal limits. X-ray detection showed normal lungs. High-resolution ultrasonography displayed normal appearance of the liver, gallbladder, pancreas, spleen, and kidneys. A 5.4 cm × 4.5 cm heterogeneous echotextured nodule in the left lobe of the thyroid was observed under ultrasonic detection, and no calcific nodule was found. No swollen lymph nodes were observed along the left carotid arteries and sternocleidomastoid.

### Pathological findings

During surgery, the intraoperative frozen section of the malignant tumor with melanin was diagnosed with a possible malignant melanoma. The left thyroid was excised. The left lobes of the thyroid were enlarged without capsules; the cut surface was brown and black. The thyroid weighed approximately 40 g. One 5.4 cm × 4 cm × 3 cm neoplasm within the left lobe of the thyroid was inspected. The neoplasm was surrounded by normal thyroid tissue. The cut surface of the neoplasm was brown, black, dark red, and partly grey white. No lymph nodes were found in the surrounding soft tissues.

**Fig. 1:**
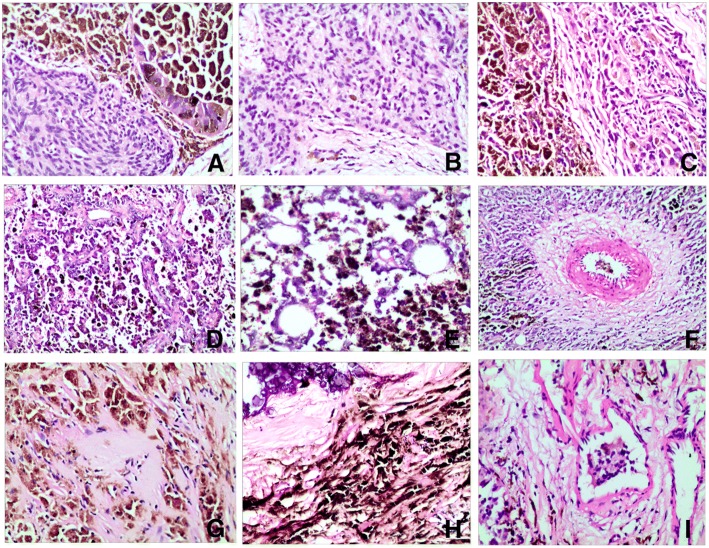
Morphological characteristics of melanin-producing MTC. H&E staining evidenced that the tumor cell with abundant black or brown melanin (**a**), minimal melanin (**b**). Melanin was found in the cytoplasm of the tumor cells or the extracellular matrix (**c**). The tumor displayed different structural morphologies: papillary (1**d**), microcapsule (1**e**), and angioneoplasm-like (1**f**). Part of the tumor cells were separated by amyloid-like material (1**g**). Psammoma bodies (1**h**) and tumor embolus (1I) was observed in certain visual fields, (hematoxylin-eosin staining, Magnification × 20)

Microscopically, the spindle, epithelioid, and polygonal tumor cells spread diffusely or storiform arranged. The tumor cells exhibited moderate-to-abundant eosinophilic red-staining cytoplasm, necrosis, and abundant black or brown melanin (Fig. 1a). Focal hemorrhage with no or minimal melanin was observed (Fig. 1b). Melanin maldistribution was noted; melanin was found in the cytoplasm of the tumor cells or the extracellular matrix (Fig. 1c). The tumor displayed different structural morphologies: widespread, nest, papillary, microcapsule, and angiomatoid structure (Fig. 1d–f). Part of the tumor cells were separated by a fibrovascular matrix and amyloid-like material (Fig. 1g). The tumor cells with melanin were moderate to large, and those without melanin were small. The tumor cells exhibited a low mitotic rate (three mitoses per 10 high-power fields (HPF)). Psammoma bodies were observed in the tumor (Fig. 1h). Tumor embolus was observed in certain visual fields (Fig. 1i).

### Immunohistochemical staining and results

Immunohistochemical staining was performed according to open-accessed data. Formalin-fixed and paraffin-embedded tumor blocks were cut into 3 μm-thick sections and immunohistochemically stained. High-pressure cooking was conducted for antigen retrieval in 10 mM citrate buffer (pH 6.0). The primary antibodies were added according to the dilution in Table 1 and incubated overnight at 4 °C. After one day, the slides were incubated with the secondary antibodies and visualized with DAB. The results of the immunochemical staining are shown in Table 1.

**Table 1:** Primary antibodies used in the study and their source

Ab	Dilution	Clone	Source
syn	1:100	Monoclonal mouse IgG	Maxim
calcitonin	1:150	Monoclonal mouse IgG	Maxim
CgA	1:50	Monoclonal rabbit IgG	Maxim
melanoma	1:200	Monoclonal mouse IgG	Maxim
CD56	1:50	Monoclonal mouse IgG (10G9)	Millipore
cyclinD1	1:200	Monoclonal mouse IgG	Maxim
TTF-1	1:200	Monoclonal mouse IgG	Maxim
S-100 protein	1:800	Monoclonal mouse IgG (8B10)	Abcam
TG	1:300	Monoclonal mouse IgG	Maxim
Napsin A	1:500	Monoclonal mouse IgG	Maxim
calretinin	1:200	Monoclonal mouse IgG	Maxim
Ki-67	1:600	Monoclonal mouse IgG	Maxim
EMA	1:800	Monoclonal mouse IgG	Maxim
AE1/AE3	1:500	Monoclonal mouse IgG	Maxim
Melan A	1:300	Monoclonal mouse IgG	Maxim
vimentin	1:200	Monoclonal mouse IgG	Maxim
HMB-45	1:500	Monoclonal mouse IgG	Maxim

*syn* synaptophysin, *CgA* chromogranin A, *TTF-1* thyroid transcription factor-1, *TG* thyroglobulin, *EMA* epithelial membrane antigen

**Fig. 2:**
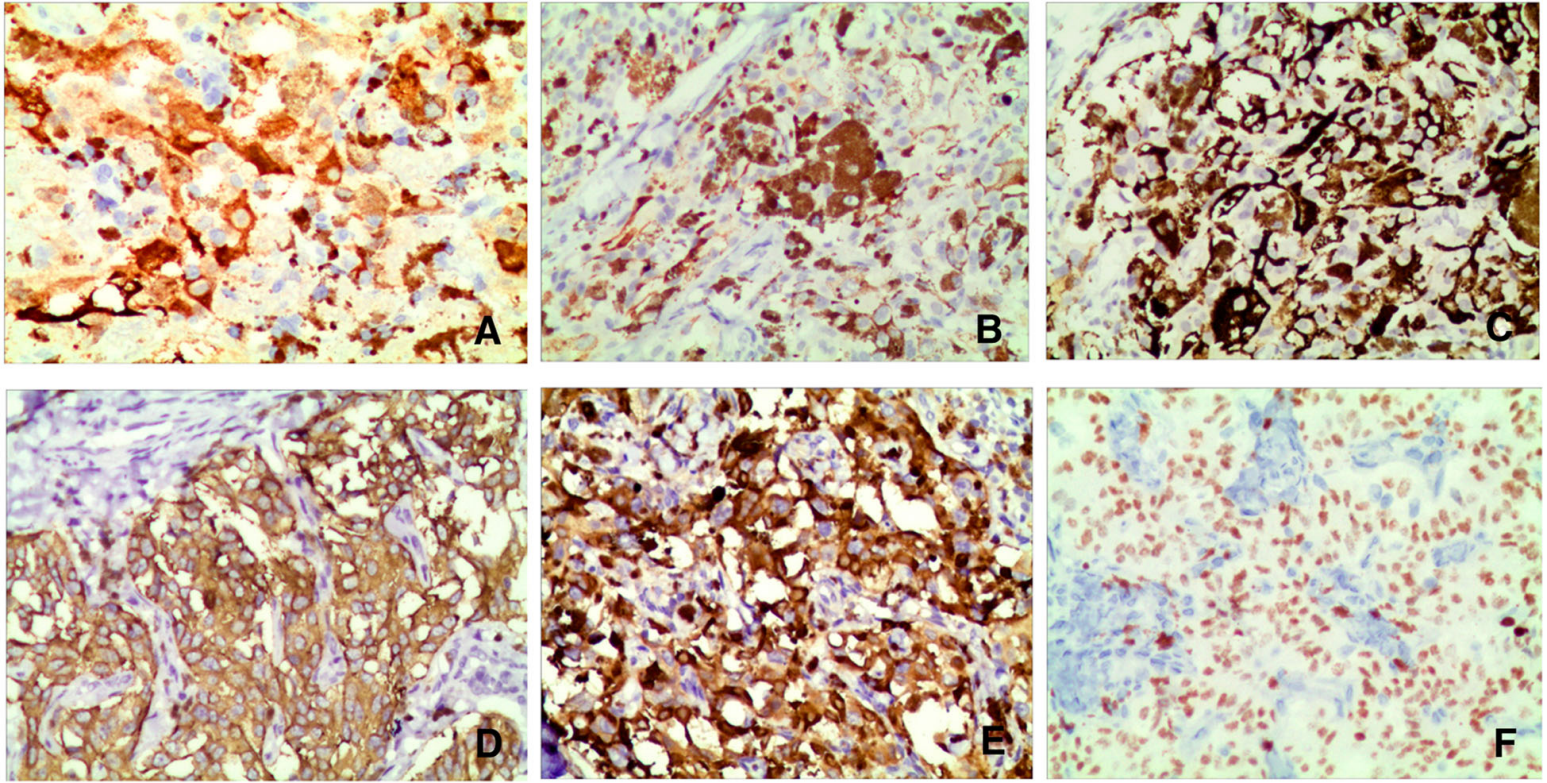
10.1186/s13000-018-0764-2The immunophenotype of melanin-producing MTC. The tumor cells were positive for S-100 protein (2**a**), melan A (2**b**), HMB-45 (2**c**), syn (2**d**), calcitonin (2**e**) and TTF-1 (2**f**). (Magnification × 20)

The tumor cells were positive for AE1/AE3, S-100 protein (Fig. 2a), melan A (Fig. 2b), HMB-45 (Fig. 2c), vimentin, synaptophysin (syn, Fig. 2d), calcitonin (Fig. 2e), chromogranin A (CgA), melanoma, cyclin D1, CD56, and, notably, thyroid transcription factor-1 (TTF-1, Fig. 2f). The neoplastic cells were negative for thyroglobulin (TG), epithelial membrane antigen, napsin A, and calretinin. The total index of ki-67 staining was approximately 30%.

### Molecular genetic detection

**Fig. 3:**
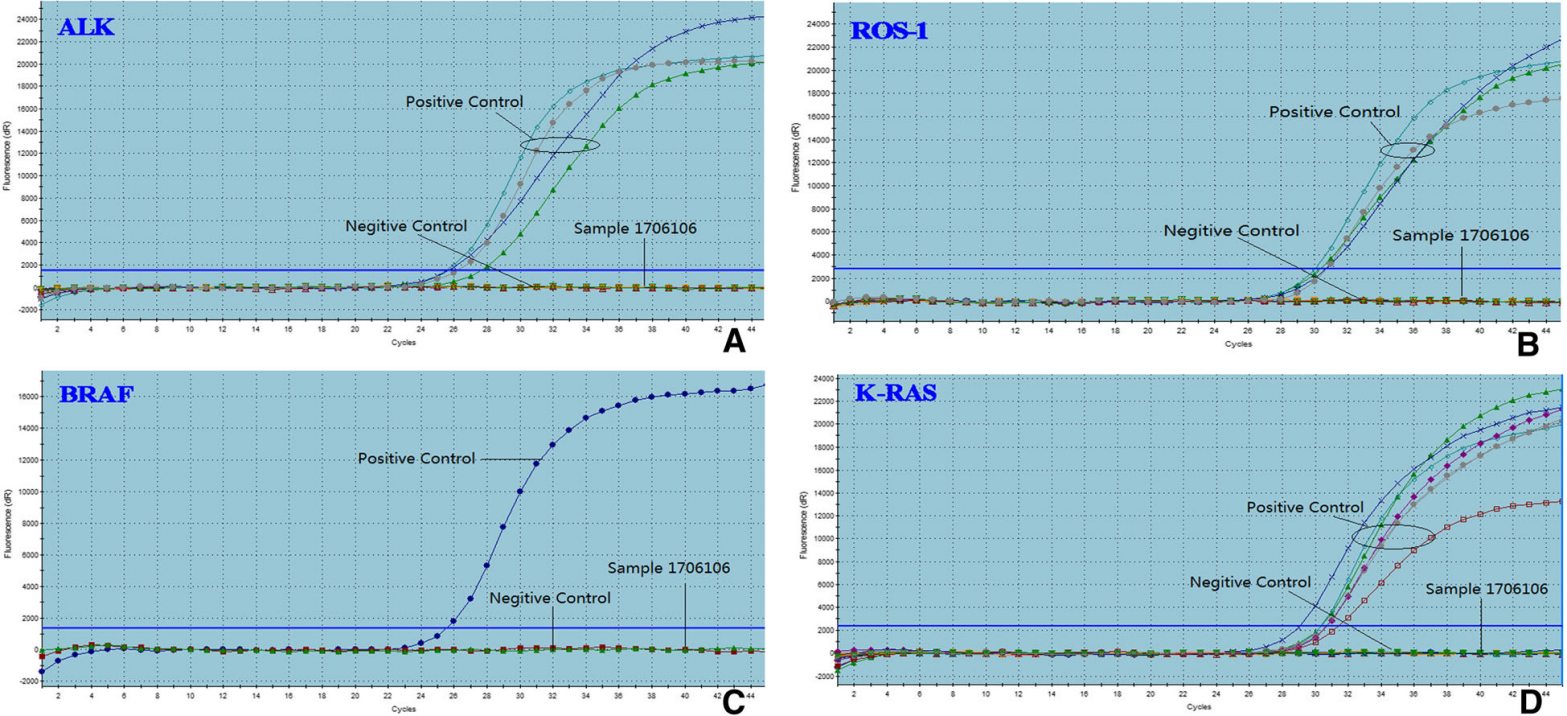
The genetic features of melanin-producing MTC. There were no mutation and gene fusion in this sample

**Fig. 4:**
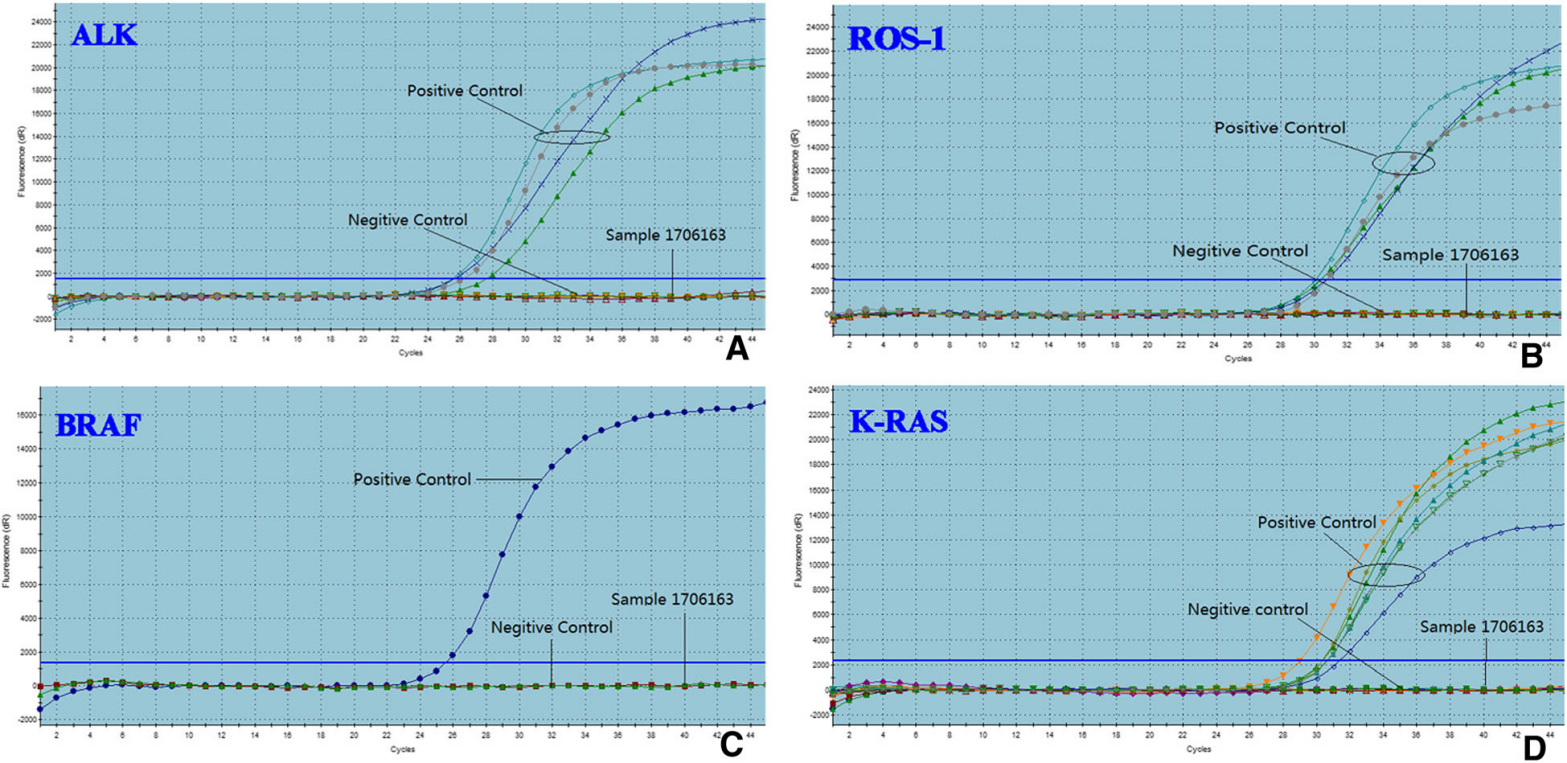
The genetic features of MTC without melanin production. There were no mutation and gene fusion in this sample

We detected BRAF V600E mutation, ROS-1 rearrangement, ALK and K-ras gene mutation in this case to study the genetic features of melanin-producing MTC (Fig. 3). Similar genetic features were analyzed in two cases of MTC without melanin production as matched control (Fig. 4). BRAF V600E mutation was detected by using the ADX-ARMS kit (AmoyDx company, Xiamen, China) according to the manufracture’ instruction. And the ROS-1 rearrangement, ALK and K-ras mutation were detected by using RT-PCR method. No mutation or gene fusion was detected in the samples.

### Treatment and follow up

Fluorine-18 fluorodeoxyglucose positron emission tomography/computed was performed after the operation to find any potential residual or metastatic foci, and the result was negative. The patient was followed up closely because no chemo- or radiotherapy was administered for the melanin-producing MCT. The patient showed no symptoms and recurrence at the 12 month follow up.

## Discussion

MTC presents a wide variety of morphological phenotypes, such as follicular, papilliferous, small cell, giant cell, clear cell, oncocytic, squamous cell, and melanotic carcinomas [2–13]. Melanin-producing MTC was reported first by J. N. Marcus [2] and is an extremely rare clinically. This type of MTC is characterized by melanin in the cytoplasm of tumor or stromal cells and amyloid in the interstitial tissue. Melanin in melanin-producing MTC ranges from scant to massive [8]. To date, 14 cases of melanin-producing MTC (including this case) have been reported in the literature. Seven cases were males, and seven were females. The ages of patients with melanin-producing MTC ranged from 20 years to 72 years (50.4 ± 15.0 years), with a broad age distribution in adults.

Melanin-producing MTC is diagnosed mainly based on the morphological features and necessary biomarkers but should be differentially diagnosed from the metastatic or primary thyroid tumors. (1) Malignant melanoma of thyroid: malignant thyroid melanoma is the most common metastatic tumor in the thyroid [8, 9]. It is difficult to differentiate from melanin-producing MTC based solely on the morphology of the tumor cell. However, the patient with metastatic melanoma presented a medical history; the primary locus is found through detailed inquisition. Immunohistochemistry distinguishes the tumors, the tumor cells of melanin-producing MTC are positive for calcitonin, the most specific marker for C cells, and confirms the melanin-producing MTC instead of metastatic melanoma. (2) Undifferentiated carcinoma of thyroid: The tumor cells of undifferentiated thyroid carcinoma are positive for TTF-1, TG, CK19 and negative for calcitonin, whereas the tumor cells of melanin-producing MTC are positive for calcitonin and TTF-1 and negative for TG and CK19. (3) Melanotic paraganglioma of thyroid: The tumor cells of melanotic thyroid paraganglioma are positive for syn and negative for CgA, pan-cytokeratin, calcitonin, and TG, and stromal cells are positive for S-100 [14]. The structural morphological characteristics of the tumor cells are different between melanotic paraganglioma and melanin-producing MTC.

A panel of primary antibodies was used to diagnose the melanin-producing MTC, but results varied. Singh K showed that the tumor cells are positive for calcitonin, HMB-45, carcinoembryonic antigen (CEA), syn, chromogranin, vimentin, and epithelial membrane antigen and negative for TG, S-100, melan A, and TTF-1 [12]. Guiping Qin demonstrated that the tumor cells are positive for vimentin, CK, CgA, syn, CEA, calcitonin, HMB-45, and S-100 and negative for TG and TTF-1 [11]. The published literature reported that the tumor cells are positive for calcitonin (100%, 11/11), HMB-45 (90.1%, 10/11), CgA (54.5%, 6/11), CEA (36.4%, 4/11), S-100 (36.4%, 4/11), and CK (36.4%, 4/11). The tumor cells are negative for TG and TTF-1. In this case, the tumor cells were positive for the abovementioned antibodies, as well as melan A, cyclin D1, and CD56. The tumor cells were also positive for TTF-1, which is a biomarker for follicular epithelium carcinoma of the thyroid. The tumor cells in our case were positive for both TTF-1 and calcitonin. This paper was the first report of such finding. The reported samples did not mention the result of TTF-1 in 10 cases; one case was negative for TTF-1.

Kimura et al. [5] showed that tumors of melanin-producing MTC are derived from cells with melanocytic and C cell characteristics, that is, the cells that produce both melanin and calcitonin. A study showed malignant melanoma originating from melanin-producing MTC [15].

Congo red staining was performed to verify the presence of amyloid materials among the nests of tumor cells, and results revealed amyloid materials in the stroma.

Owing to the low incidence, the prognosis of melanin-producing MTC is unclear clear. In the 14 cases of melanin-producing MTC, three subjects died with metastasis at 13, 20, and 24 months after surgery, respectively [6, 12], indicating a death rate of 23.1% (3/13). Satisfactory follow up information was not obtained from four cases. No evidence of recurrence or metastasis was detected in five cases at 30, 12, 12, and 12 months and 11 years after the operation ([2, 7, 9], present case, [15]). Two cases at 12 and 24 months after the operation, respectively, exhibited metastasis [5, 11]. Melanin-producing MTC may be a low-malignant tumor based on the limited data. The biobehavior and prognosis of melanin-producing MTC need to be further studied, and more cases should be retrospectively analyzed. Kamaljeet Singh showed that a high mitotic count is associated with recurrence and poor prognosis [12]. In our case, the mitotic rate was approximately 3/10 HPF, and the patient was tumor-free at the 12-month follow up.

### Conclusion

In summary, we reported the clinical characteristics, immunophenotype, and genetic features of a case of melanin-producing MTC. No typical genetic features were found. Melanin-producing MTC is a special type of MTC with an extremely low incidence and unclear biobehavior. Therefore, melanin-producing MTC should be differentiated from other neoplasms.

## Abbreviations

CEA: Carcinoembryonic antigen
CgA: Chromogranin A
HPF: High-power fields
MTC: Medullary thyroid carcinoma
syn: Synaptophysin
TG: Thyroglobulin
TTF-1: Thyroid transcription factor-1
